# Artificial Intelligence and Decision-Making in Oncology: A Review of Ethical, Legal, and Informed Consent Challenges

**DOI:** 10.1007/s11912-025-01698-8

**Published:** 2025-06-17

**Authors:** Eliza-Maria Froicu, Ioana Creangă-Murariu, Vlad-Adrian Afrăsânie, Bogdan Gafton, Teodora Alexa-Stratulat, Lucian Miron, Diana Maria Pușcașu, Vladimir Poroch, Gema Bacoanu, Iulian Radu, Mihai-Vasile Marinca

**Affiliations:** 1 Department of Medical Oncology, Regional Institute of Oncology, 700483 Iasi, Romania; 2 Department of Oncology, Faculty of Medicine, “Grigore T. Popa” University of Medicine and Pharmacy, 700115 Iasi, Romania; 3 2nd Internal Medicine Department, Faculty of Medicine, “Grigore T. Popa” University of Medicine and Pharmacy, 700115 Iasi, Romania; 4 First Surgical Oncology Unit, Department of Surgery, Regional Institute of Oncology, 700483 Iasi, Romania; 5 Department of Surgery, Faculty of Medicine, “Grigore T. Popa” University of Medicine and Pharmacy, 700115 Iasi, Romania; 6 Department of Palliative Care, Regional Institute of Oncology, 700483 Iasi, Romania; 7 Department of Medical Genetics, Faculty of Medicine “Grigore T. Popa, University of Medicine and Pharmacy, 700115 Iasi, Romania; 8 Advanced Center for Research and Development in Experimental Medicine (CEMEX), “Grigore T. Popa” Medicine and Pharmacy University, Iasi, Romania; 9 Centre for Translational Medicine, Semmelweis University, Budapest, Hungary

**Keywords:** Artificial intelligence, Decision making, Oncology, Ethical, Legal, Informed consent

## Abstract

**Purpose of Review:**

Artificial Intelligence (AI) integration in oncology is transforming therapeutic decision-making by providing clinical decision support. AI may improve treatment precision, but it raises ethical, legal, and informed consent issues. This review examines these paramount AI implementation issues in cancer care. This systematic review followed the PRISMA 2020 guidelines and was prospectively registered in the PROSPERO (CRD420251046482) database. A comprehensive literature search was conducted in PubMed, Embase, and the Cochrane CENTRAL Library to identify studies published between January 2015 and May 2025. AI-supported oncology therapeutic decision-making studies with ethical, legal, or informed consent implications were eligible.

**Recent Findings:**

Fifteen studies met the inclusion criteria. AI applications were found to support treatment recommendations, personalize drug dosing, and improve adherence and patient management. Despite these benefits, the review highlighted key concerns, including algorithmic transparency, unclear accountability in AI-guided decisions, data privacy, and gaps in patient understanding of AI’s role in their care.

**Summary:**

AI has the potential to enhance oncological care, but ethical and legal issues must be addressed for safe and equitable implementation. Emphasis should be placed on developing robust informed consent models, mitigating algorithmic bias, and establishing clear legal accountability. Future research must establish ethical frameworks and regulatory mechanisms to protect patient autonomy and responsibly integrate AI into oncology.

**Supplementary Information:**

The online version contains supplementary material available at 10.1007/s11912-025-01698-8.

## Introduction

Artificial Intelligence (AI) is a fast-evolving family of technologies with broad applicability in various fields such as economy, environmental sciences, or manufacturing. In healthcare, AI has the potential to revolutionize translational research and clinical practice by automating tasks, improving efficiency, and making more accurate predictions based on advanced data analysis [1]. These data can be genetic, proteomic, clinical, imaging (radiology, pathology, endoscopy), demographic—virtually every component of patient care that we are able to gather, measure, and repeat. Machine learning (ML) models accumulate information, detect patterns, and provide predictions or recommendations derived from input data. However, these models are only as effective as the data used to train them [2].

In addition to keeping informed about technological advancements, healthcare professionals must engage with the new and evolving ethical and legal implications of applying AI to medicine. This includes identifying, understanding and regulating issues related to data privacy, patient consent, algorithmic transparency, and accountability for decisions made by AI systems. Automated decision-making and profiling have the potential to compromise individuals'rights and freedoms, underscoring the need for the establishment of an appropriate and robust legal framework. Knowledge of these areas will enable clinicians to individually minimize the risk of harm, ensure equitable access, and protect patient rights. Similarly, patients should be made aware of their rights regarding data security and informed consent, ensuring they understand how their health data is used in treatment decisions [3].

The concept of"big data"continues to evolve [4], yet the fundamental questions it seeks to answer remain consistent: for oncologists, identifying the most effective treatments to recommend, and for patients, determining the best therapeutic strategy for their individual needs. A shared awareness of the increasing role of data-driven decisions is essential to ensure that big data is used responsibly, maintaining patient autonomy, privacy, and equality in care.

This systematic review aims to specifically examine the ethical, legal, and informed consent challenges associated with clinical trials involving AI-driven therapeutic decision-making in oncology, focusing on how these considerations affect clinicians and patients as AI becomes integral to cancer treatment.

## Methods

We have reviewed the available literature on AI models used for aiding therapeutic decisions in oncology, highlighting the ethical and legal challenges of integrating these technologies into patient care. We aimed to identify areas lacking transparency and explainability, as well as discuss the barriers hindering the ethical and patient-centered implementation of AI in oncology decision-making. Our systematic review followed the 2020 PRISMA (Preferred Reporting Items for Systematic Reviews and Meta-Analyses) checklist and guidelines (*Supplementary, Table 2*) and adhered fully to the methodology of the Cochrane Handbook (Cumpston et al., 2019). We followed our study protocol, which was initially registered on PROSPERO—CRD420251046482 [5].

### Eligibility Criteria

We conducted a systematic review of the literature published between January 2015 and May 2025 to explore how artificial intelligence (AI) technologies are applied in therapeutic decision-making for solid tumors, with particular attention to their ethical, legal, and consent-related implications. The search was intentionally broad and inclusive in terms of methodology, allowing for a comprehensive understanding of the diversity of AI interventions and their clinical and ethical ramifications. As such, we included studies employing qualitative, quantitative, or mixed-method approaches, encompassing experimental, observational, cross-sectional, and qualitative designs.

To be eligible, studies had to meet several criteria: they were required to implement at least one AI intervention—or a specific subset such as machine learning, deep learning, or natural language processing—in the context of systemic treatment decision-making in oncology. Moreover, the included studies needed to focus exclusively on cancer patients and address at least one dimension related to the ethical, legal, or informed consent aspects of using AI in therapeutic settings. Only articles written in English and available in full-text form were considered.

In contrast, we excluded studies where AI was applied solely in non-therapeutic domains such as cancer diagnosis, histopathology, imaging, surgery, or radiotherapy without reference to systemic treatment planning. Likewise, we excluded review articles, case reports, and publications limited to conference abstracts or posters, given their limited methodological transparency and peer-review status.

The overall search strategy was conducted following the PICO framework. The population (P) included cancer patients undergoing AI-supported therapeutic decision-making; the intervention (I) referred to the use of AI-based tools in medical oncology to aid treatment selection; the comparison (C) was conventional care relying on clinician-led decisions without AI input and finally the outcome (O) was defined as the clinical adoption of AI decision-support systems during oncology consultations, whereby physicians actively incorporated algorithm-generated insights into patient management.

### Search Strategy

The systematic search was conducted on 5 th of May in three databases, CENTRAL Cochrane Library, PubMed, EMBASE. The search strategy used a combination of MeSH terms arranged in the following search string: (oncology OR cancer OR oncologists) AND ("artificial intelligence"OR"machine learning"OR AI OR “deep learning”) AND (decision OR treatment OR therapy OR “treatment planning” or “personalized oncology” or “clinical trial”) AND (ethics OR"informed consent"OR legal OR “data privacy”).

### Selection Process

The selection of studies was performed independently by three reviewers (EMF, VAA, BG). All identified references were initially imported into EndNote 20 (Clarivate, 2013) to eliminate duplicates. Titles and abstracts were then screened using Rayyan (Version 1.0, Qatar Computing Research Institute, Qatar), followed by a full-text review. At each selection stage, inter-rater agreement was assessed by calculating Cohen’s kappa coefficient (κ). Any discrepancies between the three reviewers were resolved through consultation with a fourth independent reviewer (IC-M). After relevant articles were selected for inclusion in this review, the reference list and citations of each article were inspected for additional eligible publications.

### Data Collection Process

Data extraction was conducted independently by three reviewers (EMF, VAA, TAS), with disagreements adjudicated by a fourth reviewer (IC-M). A standardized data extraction sheet was employed to systematically collect key information from eligible studies, including article title, first author, year of publication, Digital Object Identifier (DOI), population demographics and particularities, AI application type (intervention), key findings: therapeutic decision-making impact (outcome), ethical, legal, and consent challenges (Table 1*; Supplementary, Table 3*).

**Table 1:** Main findings of included studies

Study	Population	AI Intervention	Key Findings (Impact)	Ethical, Legal & Consent Issues
Rinderknecht 2024 [6]	Oncologists and urogenital cancer patients using simulated cases for AI evaluation	ChatGPT-4 and Claude 3.5 Sonnet assessed via modified System Causability Scale (mSCS)	mSCS outperformed original SCS; strong reliability and validity; foundational for CONCORDIA trial	Ethics approval obtained; patient data privacy respected; potential limitations in LLM clinical nuance; informed consent risks in AI treatment advice
Curia 2021 [7]	Women at risk for cervical cancer using explainable AI risk predictions	Ensemble machine learning with LIME and SHAP for transparent interpretation	High classification accuracy; supports clinicians in understanding and justifying treatment choices	Need for interpretability to avoid black-box decisions; clinicians remain legally accountable; data privacy vs transparency tension
Parikh 2022 [8]	29 U.S. oncology clinicians evaluating prognostic algorithms in practice	ML tools for mortality prediction to guide advance care planning	Supported clinician judgment and facilitated difficult conversations; concerns about accuracy and emotional impact on patients	Ethical risks in disclosing high-risk outcomes; unclear liability when predictions guide decisions; emphasized informed use
Tan 2021 [9]	Metastatic cancer patients in Singapore undergoing capecitabine chemotherapy	CURATE.AI platform for real-time dose adjustment based on biomarkers	Feasibility confirmed; adherence and timeliness of dose changes recorded; precision oncology improved	Data stored securely; consent obtained under local and international standards; ethical adherence to trial protocols
Ng 2023 [10]	Patients with hepatobiliary tumors in multidisciplinary tumor board setting	ADBoard AI platform integrating ML and NLP for enhancing MDM efficiency	Improved concordance with MDM decisions; streamlined patient data review; improved decision transparency	Ethical need for accurate automated data extraction; compliance with data sharing regulations; full informed consent required
Masiero 2023 [11]	100 metastatic breast cancer patients divided into intervention and control groups	TREAT web platform using ML for predicting nonadherence and improving therapy adherence	Anticipated improvement in treatment adherence and patient outcomes; potential cost savings	GDPR-aligned data collection; secure digital tools; patients informed of participation rights
Tzelves 2022 [12]	Breast and prostate cancer survivors across four EU countries	ASCAPE AI platform analyzing wearables and EMR data to tailor supportive care	Enabled patient-specific rehab plans; improved engagement and quality of life	Non-invasive approach respecting autonomy; GDPR compliance ensured; opt-out options clearly defined
Lococo 2023 [13]	600 lung cancer patients from 5 European centers	Digital Human Avatars integrating omics and clinical data with ML	Enhanced diagnosis accuracy and personalized treatment; expected lower toxicity and improved cost-efficiency	Comprehensive data governance (FAIR/GDPR); multi-source consent procedures required for open data access
Aghamaliyev 2024 [14]	115 gastrointestinal cancer cases evaluated by tumor boards	ChatGPT 3.5-generated plans compared with human MTBs	83% general concordance; lower agreement in chemo/follow-up specifics	AI safety in clinical use questioned; potential risks in therapy precision; role of human oversight critical
Janbain 2024 [15]	1029 prostate cancer patients post-PSMA-PET imaging in 5 countries	Random Survival Forest model vs Cox model for predicting relapse-free survival	RSF outperformed existing tools; aided personalized salvage radiotherapy plans	Secondary data use ethically approved; data anonymized; consent waiver granted under original data terms
Lazris 2024 [16]	Cancer patients exploring symptom management options	ChatGPT-3.5 vs NCCN guidelines for supportive care (9 symptoms)	Only 37.3% agreement with NCCN; ChatGPT responses more readable but less specific	Misleading recommendations possible; patient awareness of AI limitations critical; unclear liability if harm occurs
Shimada 2023 [17]	213 terminal cancer patients in palliative care	ML model predicted hidden symptoms using decision trees and patient history	Accurate for pain, fatigue, spiritual distress; potential to support palliative care teams	Consent flagged as predicted concern; highlights vulnerabilities in end-of-life communication
Stalp 2024 [18]	30 standardized breast cancer case scenarios	ChatGPT-generated treatment plans reviewed by oncologists	Accurate in HER2 and early-stage cases; poor in complex/postoperative planning	Need for clinician validation; ethical use in low-risk contexts only; unclear medico-legal safeguards
Hesjedal 2024 [19]	Scientists, doctors, and patients in prostate cancer diagnostics	AI support tools for interpreting MRI scans in prostate cancer care	General optimism; MDs emphasize AI as assistive, not autonomous; patients trust doctors over algorithms	Clear accountability structures missing; ethical standards must be human-led; liability concerns widespread
Li 2024 [20]	228 Chinese oncologists across care levels	Opinions on AI in oncology decision-making and doctor-patient dynamics	71% feared overreliance; 54% noted bias; regulation lag widely reported	Call for updated laws and transparency; divided opinion on whether AI may replace doctors

## Results

**Fig. 1:**
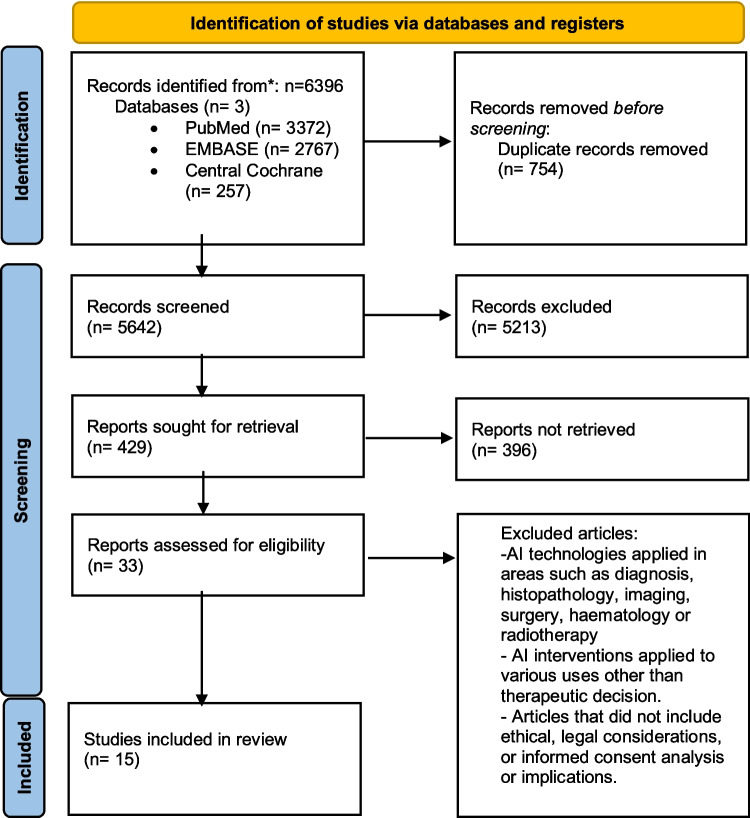
PRISMA flowchart of the article selection process

A total of 429 records were identified through the systematic literature search. Following screening and eligibility assessment, 15 studies met the inclusion criteria and were included in the final analysis [6–20]. A summary of the selection process is presented in Fig. 1.

### Characteristics of Included Studies

Out of 15 studies, both medical oncologists and patients with various types of cancer were included in the study's population. The AI intervention consisted of generative artificial intelligence such as large language models [6, 14, 16, 18], supervised machine learning for instance random survival forest [15], explainable AI techniques [7], and a machine learning–based web application [9, 10] The primary impact of therapeutic decision-making was assessed for each study, and subsequently, they were classified into thematic groups for the analysis of ethical, legal, and informed consent challenges. Of the chosen papers, all of them addresed AI-related ethical issues, but only eleven covered legal concerns [7–13, 15, 16, 19, 20], and ten the informed consent process, respectively [6, 8–13, 15–17] Table 1 summarizes all the important characteristics of included studies; *Table 3 (Supplementary)* presents all the aspects in a more detailed manner.

### Enhancing Treatment Recommendations and Decision Support

AI models have exhibited strong performance in aligning with expert recommendations for cancer treatment. In the work done by Lazris et al., 2024, ChatGPT- 3.5 provided case-specific treatment recommendations in 81% of cases, with an overall treatment strategy concordance of 83%. However, exact treatment plan concordance was lower (65%), with challenges in chemotherapy regimen recommendations and follow-up protocols. Similarly, AI-based models demonstrated high sensitivity and accuracy in classifying cancer risk, supporting clinicians in making informed treatment decisions [16].

Ng et al., 2023 described in their work how they designed ADBoard to improve decision-making in multidisciplinary meetings (MDMs). Their tool ensured the completeness of patient information and enhanced the explainability of decision protocols. The adoption of AI in MDM is expected to streamline workflow, reduce administrative burdens, and allow clinicians to focus on complex cases [10].

*Action point: Future practices should focus on integrating AI precision in chemotherapy recommendations, enhancing multidisciplinary decision transparency, and streamlining clinical workflows for oncology treatment support.*

### Optimizing Drug Dosing and Precision Medicine

AI-driven platforms have shown promise in personalized dose optimization. The CURATE.AI platform successfully provided individualized dosing recommendations, aiming to enhance therapeutic decision-making by optimizing drug administration. Tan et al. 2021 conducted a pilot study to evaluate its viability by monitoring the timeliness of dose recommendations, and the frequency of clinically significant dose modifications suggesting feasibility for broader implementation in precision oncology. Secondary outcomes included physician adherence to AI-recommended dose. Furthermore, AI-assisted molecular tumor boards (MTBs) demonstrated close alignment with ideal treatment plans (consensus annotations), often outperforming conventional MTB recommendations. The AI model facilitated standardised MTB discussions, reduced physician workload, and supported precision oncology frameworks that could be expanded globally [6, 14].

*Action point: To effectively formulate precision medicine treatment recommendations, MTBs must navigate the vast array of molecular alterations and immune markers unique to each patient's tumor. Integrating AI and ML into MTB workflows could significantly enhance accessibility, facilitate standardized implementation, and enable routine adoption even in smaller clinical centers.*

### Improving Treatment Adherence and Patient Management

Masiero et al., 2024 found that AI-driven clinical decision support systems (DSS) could improve adherence to oral anticancer treatments by predicting behavior [11]. A ML-integrated DSS was evaluated for its effectiveness in promoting medication adherence, with secondary objectives of identifying new predictive variables to refine adherence behavior models. The study anticipates that improved adherence will positively influence clinical outcomes and reduce the economic burden of nonadherence. Similarly, AI was utilized to support personalized patient follow-up strategies, enhancing engagement and optimizing clinician decision-making regarding supportive care interventions. These AI-assisted approaches are expected to improve quality of life by reducing treatment-related toxicity and unnecessary interventions [12].

Beyond direct therapeutic decision-making, AI has shown potential in predicting nonvisible symptoms relevant to palliative care. AI models achieved varying levels of accuracy (55.5%–88.0%) in predicting symptoms such as pain, dyspnea, fatigue, delirium, and anxiety. These predictive capabilities could help optimize symptom assessment and management, assisting clinicians in delivering early interventions for patients requiring supportive and palliative care [17].

*Action point: AI models could help shape multiple different patient profiles to assist the medical team in better identifying individual issues, needs and expectations that, once addressed, might improve treatment results beyond the generally predicted values obtained from clinical trials and real-world registries.*

### Challenges and Considerations in AI-Driven Therapeutic Decision-Making

While AI has demonstrated potential in optimizing therapeutic decisions, several concerns remain. Clinicians acknowledged AI’s role in validating their prognostic judgments and guiding treatment discussions but expressed reservations regarding AI accuracy, variability in patient responses, and the risk of over-reliance on algorithmic predictions [8].

According to Stalp et al., 2024, ChatGPT’s treatment recommendations were generally sufficient, with high accuracy observed in HER2-positive breast cancer cases and better performance in primary, non-complicated scenarios, particularly for chemotherapy [18]. However, its limitations became evident in complex and postoperative cases, where it struggled to provide proper chronological treatment sequences and precise recommendations, underscoring the need for human oversight in intricate therapeutic decisions.

Li et al., 2024 conducted a cross-survey study to explore the Chinese oncologists'perspectives on integrating AI into clinical practice. Cancer care providers expressed concerns about AI integration in clinical practice, particularly regarding its potential to mislead diagnoses and treatments, overreliance on its recommendations, and risks related to data security, algorithm bias, and patient privacy. Additionally, many highlighted the slow adaptation of regulations to AI advancements. Views on AI’s impact on the doctor-patient relationship were mixed, with some fearing increased disputes, while opinions on whether AI might replace physicians remained divided, with no clear consensus [20].

In their qualitative study, Hesjedal et al., 2024 emphasized various opinions regarding AI's role in therapeutic decision-making. Scientists pointed out the need for robust validation and high-quality training datasets, while clinicians underscored the necessity of human oversight in AI-generated recommendations. Patients exhibited caution, expressing trust in physicians to ensure AI's reliability and safety in clinical practice. Ethical and regulatory concerns, including data security, algorithm bias, and AI's potential impact on the doctor-patient relationship, were also highlighted [19].

Evaluations are most often carried out retrospectively on small datasets that may not accurately represent the language, demographic and epidemiological characteristics of patients encountered in clinical practice.

*Action point: Providing training and education needed for healthcare professionals to effectively utilize AI tools in oncology settings, ensuring that they have the necessary skills to interpret and implement recommendations accurately.*

### Ethical, Legal, and Informed Consent Challenges

The ethical integration of AI in oncology decision-making is complex, requiring careful attention to transparency, fairness, and interpretability. One of the most pressing ethical concerns is the"black-box"nature of many ML models, which can generate treatment recommendations without clearly explaining their reasoning. This lack of interpretability challenges the principle of patient autonomy, as clinicians may struggle to justify AI-driven decisions, and patients may find it difficult to make informed choices about their care. Additionally, bias in AI models remains a major ethical issue. Given that training data often come from specific populations, AI-driven decision support tools may not generalize well to diverse patient groups, potentially exacerbating existing healthcare disparities. If these biases are not systematically addressed, AI could reinforce inequities rather than mitigate them. Another ethical dilemma is the potential psychological impact of AI-driven prognostic tools. AI models predicting survival or treatment outcomes must be used with caution, as disclosing algorithm-derived mortality estimates to patients might cause distress or even inadvertently influence clinical decision-making in ways that deprive patients of beneficial therapies. Ensuring ethical AI deployment in oncology requires rigorous validation, continuous bias assessment, and a commitment to human oversight in decision-making.

The legal landscape surrounding AI-driven decision-making in oncology remains unclear, posing significant medico-legal risks. A central concern is liability: if an AI system provides incorrect or harmful treatment recommendations, it is unclear whether responsibility falls on the clinician using the AI, the institution implementing the technology, or the developers who trained the algorithm. Existing regulatory frameworks, including those governing AI-based medical devices, have yet to fully address these liability concerns.

Additionally, compliance with data privacy regulations, such as the General Data Protection Regulation (GDPR) and Health Insurance Portability and Accountability Act (HIPAA), presents another legal hurdle. AI systems often rely on large, multicenter datasets for training, which necessitates robust safeguards to ensure data de-identification and secure storage. However, retrospective data collection, especially across multiple institutions, complicates adherence to these regulations. Furthermore, intellectual property rights and data ownership raise legal ambiguities, particularly when AI models are trained using proprietary clinical data. Striking a balance between protecting patient data and enabling AI-driven innovation requires ongoing legal scrutiny, ensuring that regulatory adaptations keep pace with technological advancements.

The use of AI in oncology decision-making presents unique challenges to the informed consent process, as patients may not fully understand the implications of AI-driven recommendations. Traditionally, informed consent relies on clear communication of treatment options, risks, and expected outcomes. However, AI's involvement introduces an additional layer of complexity, as patients must also be informed about how AI systems function, their limitations, and the extent to which they influence clinical decisions. A key issue is the potential for"automation bias,"in which both patients and clinicians over-rely on AI recommendations without critically evaluating their validity. This phenomenon raises concerns about whether consent obtained under such circumstances truly meets ethical and legal standards. Additionally, when AI systems generate treatment plans that deviate from established guidelines, there is a need for explicit disclosure to ensure patients remain adequately informed. In clinical trials incorporating AI-assisted decision-making, standardized consent protocols must be developed to explicitly outline AI’s role and potential risks. Addressing these challenges requires educational initiatives for both clinicians and patients, ensuring that AI-driven recommendations do not undermine the principles of transparency, autonomy, and shared decision-making in oncology care.

*Action point: Discuss the challenges of obtaining informed consent from patients when using AI technologies in oncology. Propose ways to improve transparency and communication between providers and patients by informing them of the decision-making process, potential consequences, and right to contest.*

## Discussion

AI tools must be trusted and accepted by patients, doctors, health organisations, and authorities if they are to be adopted in the real world. As technology advances and new tools emerge, the responsibility for understanding and interpreting AI outputs falls straight on the practitioner. A thorough grasp of the model’s design, data sources, testing protocols, and potential biases is essential for addressing ethical dilemmas, ensuring fairness, and mitigating legal risks that may arise from AI-driven decisions. A significant and well-observed problem for machine learning systems is bias in the obtained outcomes, arising from bias in training data. AI models often underperform for underrepresented groups due to biased datasets, amplifying healthcare disparities; use of multiple diverse datasets for training, as well as deployment of bias mitigation strategies, are critical.

Combining clinical data from many patient profiles and providing users with a uniform framework and a shared understanding is one of the challenges that arise when attempting to implement AI models in therapeutic decision-making. Moreover, treatment recommendation systems are inherently more complex, as they seek to identify the optimal therapeutic approach from numerous available options by analyzing diverse input data, including structured information, free text, biological assessments, and imaging. A primary limitation of such systems, especially those trained on retrospective clinical data, is their potential to quickly become outdated in rapidly advancing disciplines like oncology, where treatment choices must consistently reflect evolving guidelines and the latest evidence-based medical research [21]. In order to accomplish this goal, it is necessary to undertake a harmonisation among the several various sources of information.

The evaluation of AI-driven follow-up strategies in oncology should encompass both patient- and physician-centric dimensions through a mixed-methods approach. From the patient perspective, assessment should focus on overall experience, satisfaction, perceived barriers and facilitators to AI-supported follow-up, and motivation for adherence. Quantitative metrics, such as engagement with digital tools (e.g., completion of self-reported assessments, duration of wearable device usage) and adherence to AI-driven recommendations, should be complemented by qualitative insights to contextualize user experiences. Additionally, longitudinal monitoring of quality of life (QoL) trajectories is essential to assess the effectiveness of AI-based follow-up interventions.

The integration of AI into oncology, particularly in therapeutic decision-making, has the potential to enhance diagnostic precision, personalize treatment plans, and improve clinical outcomes by analyzing complex datasets, including genomic, imaging, and clinical data. However, the use of AI in this context raises significant ethical, legal, and informed consent challenges that require rigorous assessment to ensure these technologies align with patient-centric care and established standards.

The ethical challenges primarily involve transparency, accountability, autonomy, and bias. AI systems often lack interpretability due to their"black box"nature, creating obstacles for clinicians to explain AI-driven recommendations to patients, and thereby impacting shared decision-making and informed consent [22–24]. Additionally, concerns about algorithmic bias and health equity are particularly pressing, as AI models trained on non-representative datasets risk exacerbating disparities in cancer care for underserved populations [24–26].

From a legal perspective, the deployment of AI in oncology introduces ambiguities surrounding liability and evolving medico-legal standards. Questions about who is responsible—clinicians, developers, or healthcare institutions—when algorithmic errors occur remain unresolved [24, 27, 28]. Furthermore, traditional informed consent models are inadequate for explaining the complexities of AI systems. Dynamic consent models, which involve ongoing patient engagement and transparency, have been proposed to address these issues but remain underexplored in practice [22, 24, 29].

Patient comprehension and trust are central to ethical AI integration. Studies indicate that while AI is valued for its diagnostic precision and ability to tailor treatments, patients often lack understanding of its role, leading to skepticism and mistrust [24, 30, 31]. Clinicians may be similarly unprepared to address the ethical and regulatory complexities posed by AI, with surveys showing a significant gap in their knowledge and confidence in safeguarding against biased or harmful outcomes [32, 33].

These findings underscore the need for interdisciplinary frameworks that address not only the technical and clinical aspects of AI but also its ethical, legal, and human factors. Existing research provides valuable theoretical insights and frameworks, such as patient-centered shared decision-making guides [30], explainable AI methodologies [22, 24] and proposals for regulatory updates [24, 27, 28] approaches in oncology in terms of clinical efficacy, adherence to ethical standards, or patient comprehension and satisfaction [32, 34, 35]. This gap underscores the urgent need for robust empirical research and standardized protocols to integrate AI into real-world oncology care responsibly.

Our study isn’t without limitations. This review is limited by the small number of eligible studies, reflecting the emerging nature of AI-driven therapeutic decision-making in oncology, with most included studies observational or exploratory, with limited prospective validation. Furthermore, the predominance of retrospective data and single-center analyses may affect generalizability. Finally, language restrictions and database selection may have excluded relevant non-English or unpublished studies.

## Key Implications for Clinical Practice and Research

- Legally define quality standards for data collection and data entry processes utilized by AI systems.
- Promote the collection of diverse patient-derived data samples, emphasizing genetic specificity and its implications for personalized treatment. Analyse case studies or instances where AI technology has been effectively incorporated into oncology practices, highlighting advantages and possible disadvantages.
- Establish legal standards for validating AI-generated conclusions, mandating a formal vetting process by a designated oversight committee (analogous to existing validation procedures for medical professionals, effectively recognizing AI as a clinical decision-maker).
- Establish the criteria for an individual's legal accountability in AI applications and propose appropriate penalties.
- Address legislative discrepancies through advocacy by professional bodies (e.g., ESMO, ASCO), recognizing that current legislative disharmony constitutes a significant barrier to clinical trial accessibility.
- Advocate for the adoption of specialized European-level legislation (preferably a directive), harmonized with extracommunity jurisdictions through international treaties, potentially under the legislative guidance of the World Health Organization (WHO).
- Establish a dedicated European authority, potentially under the European Court of Human Rights (ECHR), to protect the rights of patients treated using AI-driven clinical practices.
- Develop informed consent procedures incorporating feedback from patients and patient advocacy groups.

## Conclusion

This comprehensive review provides insights into the integration of artificial intelligence in oncology practice, focusing on its ethical implications. By examining key challenges and opportunities, it seeks to assist medical oncologists in formulating responsible and equitable AI practices that prioritize patient welfare, transparency, and accountability in medical decision-making. Integrating AI into oncology decision-making presents significant ethical, legal, and patient-informed consent challenges, with gaps in transparency, explainability, and trust identified as critical barriers, while frameworks such as dynamic consent models and explainable AI are recommended to support ethical and patient-centered implementation.

## Key References

- Hesjedal, M. B., Lysø, E. H., Solbjør, M. & Skolbekken, J.-A. Valuing good health care: How medical doctors, scientists and patients relate ethical challenges with artificial intelligence decision-making support tools in prostate cancer diagnostics to good health care. Sociol. Health Illn. 46, 1808–1827 (2024).
  - This study emphasises the importance of high-quality training datasets and human oversight in AI-generated recommendations.
- Shimada, K. & Tsuneto, S. Novel method for predicting nonvisible symptoms using machine learning in cancer palliative care. *Sci. Rep.***13**, 12088 (2023).
  - AI models have demonstrated potential in predicting nonvisible symptoms related to palliative care.
- Curia, F. Cervical cancer risk prediction with robust ensemble and explainable black boxes method. *Health Technol (Berl)* **11**, 875–885 (2021). AI models have shown promising results in improving the quality of life for cancer patients receiving palliative care. tab
  - The proposed model integrates advanced ensemble techniques with explainable AI tools (e.g., LIME and Shapley) to provide transparent and interpretable risk predictions.
- Parikh, R. B. et al*.* Clinician perspectives on machine learning prognostic algorithms in the routine care of patients with cancer: a qualitative study. *Support. Care Cancer* **30**, 4363–4372 (2022).
  - A a qualitative study that explored the perspectives of clinicians on the use of machine learning prognostic algorithms in routine care for cancer patients.
- Tzelves, L. et al. Artificial intelligence supporting cancer patients across Europe-The ASCAPE project. PLoS ONE 17, e0265127 (2022).
  - The ASCAPE project is a large study conducted in four centres across Europe to investigate the potential benefits and challenges of using machine learning prognostic algorithms in cancer survivors.
- Lococo, F. et al. Lung cancer multi-omics digital human avatars for integrating precision medicine into clinical practice: the LANTERN study. BMC Cancer 23, 540 (2023).
  - This study enrols an extensive number of patients (600 lung cancer patients), which will allow for a comprehensive analysis of prognostic algorithms in this type of cancer.

## Supplementary Information

Below is the link to the electronic supplementary material.

Supplementary file1 (DOCX 51 KB)

## Data Availability

No datasets were generated or analysed during the current study.
