# Supplementary material for: Artificial Intelligence and Decision-Making in Oncology: A Review of Ethical, Legal, and Informed Consent Challenges
Source: Curr Oncol Rep. 2025 Jun 17;27(8):1002–12. doi: 10.1007/s11912-025-01698-8 (PMC12423120; doi:10.1007/s11912-025-01698-8)
Supplement: Supplementary file 1 — Supplementary file1 (DOCX 51 KB) [file 11912_2025_1698_MOESM1_ESM.docx]

**Artificial Intelligence and Decision-Making in Oncology: A Review of Ethical, Legal, and Informed Consent Challenges- *Supplementary file***

Eliza-Maria Froicu^2,3^, Ioana Creangă-Murariu^1,2,8,9*^, Vlad-Adrian Afrăsânie^1,2^, Bogdan Gafton^1,2^**,** Teodora Alexa-Stratulat^1,2,8^, Lucian Miron^1,2^, Diana Maria Pușcașu^1,7^, Vladimir Poroch^3,6^, Gema Bacoanu^3,6^, Iulian Radu^4,5^ and Mihai- Vasile Marinca^1,2^

**Affiliations**

1. Department of Medical Oncology, Regional Institute of Oncology, 700483 Iasi, Romania.
2. Department of Oncology, Faculty of Medicine, "Grigore T. Popa" University of Medicine and Pharmacy, 700115 Iasi, Romania.
3. 2nd Internal Medicine Department, Faculty of Medicine, "Grigore T. Popa" University of Medicine and Pharmacy, 700115 Iasi, Romania.
4. First Surgical Oncology Unit, Department of Surgery, Regional Institute of Oncology, 700483 Iasi, Romania.
5. Department of Surgery, Faculty of Medicine, "Grigore T. Popa" University of Medicine and Pharmacy, 700115 Iasi, Romania.
6. Department of Palliative Care, Regional Institute of Oncology, 700483 Iasi, Romania.
7. Department of Medical Genetics, Faculty of Medicine “Grigore T. Popa” University of Medicine and Pharmacy, 700115 Iasi, Romania
8. Advanced Center for Research and Development in Experimental Medicine (CEMEX), “Grigore T. Popa” Medicine and Pharmacy University Iasi, Romania
9. Centre for Translational Medicine, Semmelweis University, Budapest, Hungary

*Corresponding author: Ioana Creanga-Murariu, ioana.creanga@d.umfiasi.ro

| **Section and Topic** | **Item #** | **Checklist item** | **Location where item is reported** |
| --- | --- | --- | --- |
| **TITLE** | | |  |
| Title | 1 | Identify the report as a systematic review. | 1 |
| **ABSTRACT** | | |  |
| Abstract | 2 | See the PRISMA 2020 for Abstracts checklist. | 1 |
| **INTRODUCTION** | | |  |
| Rationale | 3 | Describe the rationale for the review in the context of existing knowledge. | 2 |
| Objectives | 4 | Provide an explicit statement of the objective(s) or question(s) the review addresses. | 2 |
| **METHODS** | | |  |
| Eligibility criteria | 5 | Specify the inclusion and exclusion criteria for the review and how studies were grouped for the syntheses. | 3 |
| Information sources | 6 | Specify all databases, registers, websites, organisations, reference lists and other sources searched or consulted to identify studies. Specify the date when each source was last searched or consulted. | 4 |
| Search strategy | 7 | Present the full search strategies for all databases, registers and websites, including any filters and limits used. | 4 |
| Selection process | 8 | Specify the methods used to decide whether a study met the inclusion criteria of the review, including how many reviewers screened each record and each report retrieved, whether they worked independently, and if applicable, details of automation tools used in the process. | 4 |
| Data collection process | 9 | Specify the methods used to collect data from reports, including how many reviewers collected data from each report, whether they worked independently, any processes for obtaining or confirming data from study investigators, and if applicable, details of automation tools used in the process. | 4 |
| Data items | 10a | List and define all outcomes for which data were sought. Specify whether all results that were compatible with each outcome domain in each study were sought (e.g. for all measures, time points, analyses), and if not, the methods used to decide which results to collect. | 4 |
|  | 10b | List and define all other variables for which data were sought (e.g. participant and intervention characteristics, funding sources). Describe any assumptions made about any missing or unclear information. | 4 |
| Study risk of bias assessment | 11 | Specify the methods used to assess risk of bias in the included studies, including details of the tool(s) used, how many reviewers assessed each study and whether they worked independently, and if applicable, details of automation tools used in the process. | NA |
| Effect measures | 12 | Specify for each outcome the effect measure(s) (e.g. risk ratio, mean difference) used in the synthesis or presentation of results. | NA |
| Synthesis methods | 13a | Describe the processes used to decide which studies were eligible for each synthesis (e.g. tabulating the study intervention characteristics and comparing against the planned groups for each synthesis (item #5)). | 4 |
|  | 13b | Describe any methods required to prepare the data for presentation or synthesis, such as handling of missing summary statistics, or data conversions. | NA |
|  | 13c | Describe any methods used to tabulate or visually display results of individual studies and syntheses. | 4 |
|  | 13d | Describe any methods used to synthesize results and provide a rationale for the choice(s). If meta-analysis was performed, describe the model(s), method(s) to identify the presence and extent of statistical heterogeneity, and software package(s) used. | NA |
|  | 13e | Describe any methods used to explore possible causes of heterogeneity among study results (e.g. subgroup analysis, meta-regression). | NA |
|  | 13f | Describe any sensitivity analyses conducted to assess robustness of the synthesized results. | NA |
| Reporting bias assessment | 14 | Describe any methods used to assess risk of bias due to missing results in a synthesis (arising from reporting biases). | NA |
| Certainty assessment | 15 | Describe any methods used to assess certainty (or confidence) in the body of evidence for an outcome. | NA |
| **RESULTS** | | |  |
| Study selection | 16a | Describe the results of the search and selection process, from the number of records identified in the search to the number of studies included in the review, ideally using a flow diagram. | 4-5 |
|  | 16b | Cite studies that might appear to meet the inclusion criteria, but which were excluded, and explain why they were excluded. | 4 |
| Study characteristics | 17 | Cite each included study and present its characteristics. | 4-5 |
| Risk of bias in studies | 18 | Present assessments of risk of bias for each included study. | NA |
| Results of individual studies | 19 | For all outcomes, present, for each study: (a) summary statistics for each group (where appropriate) and (b) an effect estimate and its precision (e.g. confidence/credible interval), ideally using structured tables or plots. | 5 |
| Results of syntheses | 20a | For each synthesis, briefly summarise the characteristics and risk of bias among contributing studies. | 6-9 |
|  | 20b | Present results of all statistical syntheses conducted. If meta-analysis was done, present for each the summary estimate and its precision (e.g. confidence/credible interval) and measures of statistical heterogeneity. If comparing groups, describe the direction of the effect. | 6-9 |
|  | 20c | Present results of all investigations of possible causes of heterogeneity among study results. | NA |
|  | 20d | Present results of all sensitivity analyses conducted to assess the robustness of the synthesized results. | NA |
| Reporting biases | 21 | Present assessments of risk of bias due to missing results (arising from reporting biases) for each synthesis assessed. | NA |
| Certainty of evidence | 22 | Present assessments of certainty (or confidence) in the body of evidence for each outcome assessed. | NA |
| **DISCUSSION** | | |  |
| Discussion | 23a | Provide a general interpretation of the results in the context of other evidence. | 10-11 |
|  | 23b | Discuss any limitations of the evidence included in the review. | 11 |
|  | 23c | Discuss any limitations of the review processes used. | 11 |
|  | 23d | Discuss implications of the results for practice, policy, and future research. | 12 |
| **OTHER INFORMATION** | | |  |
| Registration and protocol | 24a | Provide registration information for the review, including register name and registration number, or state that the review was not registered. | 3 |
|  | 24b | Indicate where the review protocol can be accessed, or state that a protocol was not prepared. | 3 |
|  | 24c | Describe and explain any amendments to information provided at registration or in the protocol. | NA |
| Support | 25 | Describe sources of financial or non-financial support for the review, and the role of the funders or sponsors in the review. | 15 |
| Competing interests | 26 | Declare any competing interests of review authors. | 15 |
| Availability of data, code and other materials | 27 | Report which of the following are publicly available and where they can be found: template data collection forms; data extracted from included studies; data used for all analyses; analytic code; any other materials used in the review. | 15 |

**Supplementary Table S2.** PRISMA Checklist.

| **Author** | **Title** | **Population (P)** | **AI application (Intervention)** | **Key findings (outcome)- Therapeutic Decision-Making Impact** | **Ethical, Legal, and Consent Considerations** |
| --- | --- | --- | --- | --- | --- |
| Rinderknecht 2024 | Modification and Validation of the System Causability Scale Using AI-Based Therapeutic Recommendations for Urological Cancer Patients: A Basis for the Development of a Prospective Comparative Study | Urogenital cancer patients, oncologists | Integration of Large Language Models (LLMs), specifically ChatGPT-4 and Claude 3.5 Sonnet, to generate therapeutic recommendations for urological cancer patients; validation and modification of the System Causability Scale (SCS) to assess the quality of AI recommendations compared to those from multidisciplinary tumor boards (MTBs) | Both the original SCS and its modified version (mSCS) demonstrated strong validity, reliability (Cohen’s K > 0.74), and internal consistency (Cronbach’s Alpha > 0.9), with the mSCS showing superior performance (p < 0.01). Two Delphi processes were used to select the LLMs and set an acceptable non-inferiority margin between AI and MTB recommendations. This work establishes the methodological prerequisites for the upcoming CONCORDIA trial, which will compare blinded mSCS assessments of MTB versus LLM recommendations using 110 urological cancer scenarios. | Ethical: • The study is ethics-approved, but real patient cases were not used due to data privacy regulations, necessitating the creation of realistic simulated cases.  Consent: • Limitations include potential issues when LLM recommendations lack clinical nuance or specificity, raising questions about informed consent for AI-generated treatment advice in complex scenarios.nal SCS and its modified version (mSCS) demonstrated strong validity, reliability (Cohen’s K > 0.74), and internal consistency (Cronbach’s Alpha > 0.9), with the mSCS showing superior performance (p < 0.01). Two Delphi processes were used to select the LLMs and set an acceptable non-inferiority margin between AI and MTB recommendations. This work establishes the methodological prerequisites for the upcoming CONCORDIA trial, which will compare blinded mSCS assessments of MTB versus LLM recommendations using 110 urological cancer scenarios. |
| Curia 2021 | Cervical cancer risk prediction with robust ensemble and explainable black boxes method | Female patients at risk for cervical cancer | Development of a robust ensemble machine learning model for predicting cervical cancer risk. The model integrates advanced ensemble techniques with explainable AI tools (e.g., LIME and Shapley) to provide transparent and interpretable risk predictions. | The proposed model demonstrates promising accuracy and sensitivity in classifying cervical cancer risk. Its explainable outputs are designed to offer clear, understandable recommendations that can be integrated into clinical decision-making, thereby aiding therapeutic planning and patient management. | Ethical: • Need for transparency and interpretability to overcome the “black-box” nature of advanced ML methods.  Legal: • Balancing data privacy and protection of intellectual property with the requirement for openness in clinical decision-making; ensuring that clinicians can be held accountable for AI-assisted decisions. |
| Parikh 2022 | Clinician perspectives on machine learning prognostic algorithms in the routine care of patients with cancer: a qualitative study | 29 oncology clinicians (19 physicians, 10 advanced practice providers) from 6 practice sites (1 tertiary, 5 community) in the USA; 14 had previous exposure to a machine learning prognostic algorithm | Machine learning prognostic algorithms designed to predict mortality risk in cancer, intended to prompt advance care planning and validate clinical intuition | Clinicians recognized the utility of such algorithms for confirming their own prognostic judgments and initiating discussions about patient goals and preferences. - They expressed concerns about the accuracy of algorithm predictions, particularly due to variability in patient responses and potential data quality issues. - There was notable apprehension regarding over-reliance on these tools, which could reduce the human element in care. - Tolerance for false positive versus false negative predictions varied significantly among participants. | Ethical: • Concerns about the ethical implications of disclosing mortality predictions to patients, including potential mental harm and misinterpretation (e.g., the risk that a high mortality prediction might unduly dissuade clinicians from offering beneficial therapies).  Legal: • Uncertainty regarding accountability and liability if algorithm-driven decisions result in adverse outcomes, given that clinical judgment is partly supplanted by algorithm outputs.  Consent: • The need for transparent communication about the use and limitations of prognostic algorithms, ensuring patients are adequately informed about how these tools influence their care and that their consent is obtained under clear, understandable terms. |
| Tan 2021 | Personalised, Rational, Efficacy-Driven Cancer Drug Dosing via an Artificial Intelligence SystEm (PRECISE): A Protocol for the PRECISE CURATE.AI Pilot Clinical Trial | Adults with metastatic solid tumours and elevated baseline tumour marker levels, planned for palliative-intent, capecitabine-based chemotherapy in Singapore | A small-data, personalised dosing platform that uses individual tumour marker levels and administered drug doses to generate efficacy-driven chemotherapy dosing recommendations | The primary outcome is the proportion of participants in whom CURATE.AI can be successfully applied to provide personalised dosing. Secondary outcomes include the timeliness of dose recommendations, adherence to CURATE.AI-recommended doses by both participants and physicians, and the proportion of clinically significant dose changes. The pilot aims to gather key feasibility data to inform a future RCT, potentially improving therapeutic decision-making in precision oncology by optimising drug dosing. | Ethical & Legal: • Patient data will be de-identified via unique patient numbers (UPNs) and stored in a REDCap database on password-protected computers, in compliance with the Personal Data Protection Act 2012. • Only authorised personnel will have access, and data will be maintained for a minimum of 6 years as per ICH GCP guidelines. As a clinical trial, it adheres to ethical committee approval, requiring informed consent from participants and compliance with local regulatory standards. Standard challenges—such as ensuring patient data privacy, maintaining transparency in AI-driven dosing recommendations, and safeguarding patient safety—are implicitly managed within the trial’s ethical framework. Consent: • Written consent will be obtained per SGGCP guidelines and the Declaration of Helsinki. Patients will receive comprehensive study information in an understandable language and are informed of their right to withdraw at any time without penalty. |
| Ng 2023 | Concordance of a decision algorithm and multidisciplinary team meetings for patients with liver cancer—a study protocol for a randomized controlled trial | Patients with hepatobiliary tumors meeting inclusion criteria for tumor board discussion at a single center | ADBoard (Therapeutic Assistance and Decision algorithms for hepatobiliary tumor Boards) aims to improve the MDM process for patients with liver cancer by using different artificial intelligence (AI) methods from the fields of natural language processing (NLP) and machine learning (ML). | The study hypothesizes that ADBoard will yield high concordance with conventional MDM recommendations (target Cohen’s kappa ≥ 0.75), improve the completeness of patient information presented, and enhance the explainability of decision-making protocols (as measured by the System Causability Scale). It is anticipated that ADBoard will streamline the MDM process by reducing administrative workload and allowing clinicians to focus on more complex cases. | Ethical: • Ensuring that automated data extraction accurately captures all relevant patient information without compromising patient safety.  Legal: • Obtaining the proper permissions and network access (e.g., from external partners like DFKI) to access the Health Data Platform in compliance with data protection regulations.  Consent: • Securing informed consent for the use of patient data and maintaining confidentiality when integrating data into the ADBoard system; addressing limitations in capturing subjective or psychosocial factors not documented in the hospital information system. |
| Masiero 2023 | A Machine Learning Model to Predict Patients’ Adherence Behavior and a Decision Support System for Patients With Metastatic Breast Cancer: Protocol for a Randomized Controlled Trial | 100 patients with metastatic breast cancer recruited at the European Institute of Oncology (IEO); 50 patients assigned to the experimental group (exposed to the intervention) and 50 to the control group | A web-based Decision Support System (DSS) named TREAT, featuring four sections (Metastatic Breast Cancer, Adherence to Cancer Therapies, Promoting Adherence, and My Adherence Diary) integrated with a machine learning–based web application. The system predicts risk factors for nonadherence by analyzing physical, psychological, social, and behavioral variables, as well as quality-of-life data collected via standardized self-reports and weekly medication diaries. | Primary Outcome: Evaluate the effectiveness of the DSS and ML application in promoting adherence to oral anticancer treatments. Secondary Outcome: Collect new predictive variables that could refine the ML model for predicting adherence behavior. While recruitment began in May 2023 (expected to conclude December 2023), the anticipated impact is an improvement in medication adherence, which is expected to positively influence clinical outcomes and reduce the economic burden of nonadherence. | Ethical:  • The study is approved by the IEO ethics committee (R1786/22-IEO1907) and adheres to relevant ethical, legal, and data protection regulations.  Legal  • Patient data, including self-reports and medication diaries, are collected via secure, web-based systems with appropriate de-identification measures.ccording to the General Data Protection Regulation (Regulation EU, 2016/679) Consent: • Written informed consent is obtained following ethical guidelines, ensuring patients understand study procedures and their rights, including the right to withdraw without penalty. |
| Tzelves 2022 | Artificial intelligence supporting cancer patients across Europe—The ASCAPE project | Breast and prostate cancer survivors from four European study sites | AI platform integrating patient data (from medical records, questionnaires, apps, wearables) to predict QoL issues and suggest tailored interventions for cancer rehabilitation | Aims to improve QoL by enabling personalized follow-up strategies, enhancing patient engagement, and informing clinician decision-making regarding supportive care | Ethical: The ASCAPE trial ensures ethical integrity by implementing only non-invasive interventions that align with standard clinical practice and are applied through shared decision-making. This guarantees patient autonomy and eliminates the risk of direct physical harm from AI-supported follow-up care. Additionally, the project places a strong emphasis on transparency and respect for participants by clearly informing them about the study's aims and procedures. Participants retain full autonomy, including the right to withdraw consent at any time through an accessible and well-defined process.  Legal: The ASCAPE project is fully aligned with the General Data Protection Regulation (GDPR), particularly Article 5, by designing its data architecture around principles of lawfulness, fairne ss, and transparency. It ensures that data processing remains purpose-specific, minimized, accurate, and securely retained only for as long as necessary. Consent: Participants will receive clear, accessible information about the research goals, data processing, and their rights. They are entitled to withdraw consent at any time through a straightforward withdrawal procedure,  Furthermore, personal data is never transferred outside the EU without appropriate safeguards, and processing is always based on explicit participant consent. This legal framework guarantees participant rights and compliance with both European and national data protection standards. |
| Lococo 2023 | Lung cancer multi-omics digital human avatars for integrating precision medicine into clinical practice: the LANTERN study | 600 lung cancer patients recruited prospectively across five European centers; involvement of multidisciplinary clinical and scientific stakeholders | Digital Human Avatars (DHA): Integration of clinical variables with multi-omics data (genomic, quantitative imaging, etc.) using advanced AI/ML techniques. | Aims to enhance therapeutic decision-making by improving diagnostic accuracy and personalizing treatment pathways. Expected outcomes include reduced overtreatment and toxicity, shortened recovery and hospitalization times, decreased healthcare costs, and overall improved patient quality of life through precise and efficient clinical models. | Ethical: The LANTERN project addresses key ethical issues such as respect for human dignity, fair distribution of research benefits and burdens, and the protection of participants' rights and interests. Special emphasis is placed on ethical oversight throughout the project lifecycle, particularly concerning the responsible development and use of AI systems in accordance with the Declaration of Helsinki and GDPR. Legal: A comprehensive Data Management Plan (DMP) is implemented to comply with GDPR and adhere to FAIR principles. Challenges include ensuring data privacy and security, obtaining informed consent for multi-source data, managing open data access with strict monitoring, and meeting legal requirements for data governance and regulatory compliance. Consent: All participants in the LANTERN project will be thoroughly informed about the nature, aims, and procedures of the study. Consent will be obtained through a clear, comprehensive process involving a signed informed consent form, ensuring participants understand their rights and the use of their data. |
| Aghamaliyev 2024 | ChatGPT's Gastrointestinal Tumor Board Tango: A limping dance partner? | 115 cases of gastrointestinal malignancies presented at MTBs | ChatGPT 3.5 was used to generate treatment recommendations based on patient data provided by a senior resident; these recommendations were compared with those from MTBs. | ChatGPT provided case-specific treatment recommendations in 81% of cases, with overall treatment strategy concordance at 83% for those cases. However, exact treatment plan concordance was 65%, with lower precision observed for chemotherapy regimens and follow-up protocols. | Ethical: Ethical concerns include the risk of imprecise recommendations impacting patient safety; challenges in replicating exact treatment plans (especially chemotherapy and follow-up) raise issues related to clinical liability and the appropriateness of using ChatGPT directly for clinical decision-making in MTBs. |
| Janbain 2024 | A Machine Learning Approach for Predicting Biochemical Outcome After PSMA-PET-Guided Salvage Radiotherapy in Recurrent Prostate Cancer After Radical Prostatectomy: Retrospective Study | 1029 prostate cancer patients undergoing salvage radiotherapy following PSMA-PET-based assessment for PSA persistence or recurrence (data collected from 13 medical facilities across 5 countries: Germany, Cyprus, Australia, Italy, and Switzerland; median age ~70 years) | Developed a Random Survival Forest (RSF) model to predict freedom from biochemical failure after salvage radiotherapy; utilized k-fold cross-validation for hyperparameter tuning and statistical performance evaluation (Harrell C-index, Brier score); compared RSF with a Cox model and an existing nomogram. | The RSF model demonstrated robust predictive performance (Harrell C-index range: 0.54–0.91) across training, testing, and external validation datasets, outperforming a previously published nomogram; its predictive accuracy may assist clinicians in tailoring treatment decisions for recurrent prostate cancer. | Ethical: The study complied with ethical standards at all participating centers, with formal approval obtained from each institutional ethics board. The retrospective design of the study minimized patient risk, and ethical integrity was maintained by following guidelines for the secondary use of clinical data. Legal: Data handling respected privacy regulations, with deidentification ensuring compliance with data protection norms. Consent: Due to the study’s retrospective nature and existing ethical approval, informed consent was waived. However, original informed consent obtained during primary data collection explicitly allowed for secondary data use, aligning with ethical and legal expectations for consent in observational research. |
| Lazris 2024 | AI-Generated Content in Cancer Symptom Management: A Comparative Analysis Between ChatGPT and NCCN | Cancer patients seeking supportive care for cancer-related symptoms (as represented by NCCN guidelines) | ChatGPT-3.5 was used to generate treatment recommendations for nine cancer-related symptoms by entering queries (e.g., "How can I reduce my cancer-related [symptom]?"). These recommendations were compared with those provided by the National Comprehensive Cancer Network (NCCN) for supportive and palliative care. | Mean percent agreement between ChatGPT and NCCN recommendations was 37.3% (range 16.7%-81.8%). - NCCN provided more specific medication recommendations, whereas ChatGPT occasionally suggested medications not mentioned in NCCN (notably in constipation and diarrhea sections). - Significant differences were observed in word count and readability (Flesch-Kincaid Grade Level), with ChatGPT responses being more concise and having a lower reading level. | Ethical: • Discrepancies between AI-generated content and evidence-based guidelines may mislead patients and affect care quality. Legal: • Potential liability issues if patients act on inaccurate or non-evidence-based recommendations provided by AI. Consent: • Patients may be unaware of the limitations and discrepancies in AI-generated advice versus professional guidelines, raising concerns over informed decision-making. |
| Shimada 2023 | Novel Method for Predicting Nonvisible Symptoms Using Machine Learning in Cancer Palliative Care | 213 end-of-life cancer patients receiving palliative care | Machine learning using decision tree analysis to predict nonvisible symptoms based on patient background data and reported visible symptoms | The model predicted nonvisible symptoms—including pain, dyspnea, fatigue, drowsiness, anxiety, delirium, inadequate informed consent, and spiritual issues—with accuracy ranging from 55.5% to 88.0%, sensitivity from 3.3% to 84.9%, and specificity from 24.1% to 96.7%. This approach has the potential to enhance symptom assessment and management in palliative care by approximating the evaluation capabilities of healthcare professionals. | Ethical: The inclusion of "inadequate informed consent" as a predicted symptom underscores challenges in patient communication and decision-making at end-of-life.  Legal: Although not explicitly discussed, the reliance on AI for symptom assessment in vulnerable populations raises questions regarding accountability and clinical liability.  Consent: Ensuring that AI predictions are interpreted correctly is critical, as misinterpretation might exacerbate issues related to informed consent and patient autonomy in sensitive care settings. |
| Stalp 2024 | Quality of ChatGPT-Generated Therapy Recommendations for Breast Cancer Treatment in Gynecology | 30 breast cancer cases | ChatGPT (version unspecified, likely 3.5) used to generate therapy recommendations for breast cancer treatment; standardized patient details and treatment options were provided as input; outputs were evaluated by oncologists using a structured questionnaire | Overall, ChatGPT’s recommendations were rated as sufficient with minor limitations. - Best performance was observed in the HER2 treatment category with high accuracy. - Primary (non-complicated) cases received more accurate recommendations, especially regarding chemotherapy. - Challenges were noted in complex cases and postoperative scenarios, particularly in providing proper chronological treatment sequences and precise recommendations. | Ethical: • Need to address ethical intricacies in relying on AI-generated treatment advice for patient care. • Balancing AI recommendations with expert clinical insights to ensure patient safety. Legal: • Although not explicitly discussed, safe implementation is implied to be crucial to avoid potential medico-legal liability. Consent: • Ensuring that patients are informed about the use of AI in treatment planning is critical, even if not directly addressed; refining input data and addressing limitations are necessary steps for responsible integration. |
| Hesjedal 2024 | Valuing good health care: How medical doctors, scientists and patients relate ethical challenges with artificial intelligence decision-making support tools in prostate cancer diagnostics to good health care | Three stakeholder groups: • Scientists developing AI decision-support tools for interpreting prostate cancer MRI scans • Medical doctors (MDs) working in prostate cancer care • Prostate cancer patients | AI decision-making support tools in prostate cancer diagnostics (with a focus on how these tools are perceived and evaluated in terms of contributing to "good health care") | • Scientists: Emphasize the need for robust, representative data and rigorous testing/validation of AI models to ensure accuracy and accountability. • MDs: Express optimism about AI’s future potential to enhance diagnostic accuracy while underscoring that AI should support rather than replace human expertise, especially in complex cases. • Patients: Place high trust in MDs and are cautious about AI, relying on professionals to ensure that AI tools are thoroughly tested. | Ethical: • Accountability: Good health care requires that AI-driven decisions be made by identifiable humans who can be held responsible. • Human qualities (ethics/morals): AI lacks inherent ethical attributes, making the transfer of ethical standards from developers to AI critical.  Legal: • Ambiguity in liability: Unclear legal frameworks regarding who is responsible for AI-based decisions, with concerns about potential medico-legal consequences if AI recommendations lead to errors. |
| Li 2024 | Chinese Oncologists' Perspectives on Integrating AI into Clinical Practice: Cross-Sectional Survey Study | 228 Chinese oncologists | Exploration of oncologists' concerns regarding the integration of AI into clinical practice, specifically addressing its impact on diagnostic and treatment decision-making, doctor-patient relationships, and the potential for AI to replace physicians. | 1.5% expressed concern that AI could mislead diagnosis and treatment. - 71% worried about overreliance on AI. - 54% were concerned about data and algorithm bias, as well as issues with data security and patient privacy. - 50.4% noted a lag in the adaptation of laws, regulations, and policies relative to AI’s development. - Mixed views on AI's impact on the doctor-patient relationship (53.1% positive, 35.5% uncertain, 9.2% fearing increased disputes). - Opinions on whether AI will replace doctors were diverse, with no clear consensus. | Ethical: • Necessity for transparency in AI systems to ensure trust and uphold human-centered care. • Emphasis on bias mitigation and maintaining high standards in clinical decision-making.  Legal: • Concerns over data security and patient privacy in the absence of updated regulatory frameworks. • Lagging legal, regulatory, and policy adaptations to match AI development in healthcare. |
| Rinderknecht 2024 | Modification and Validation of the System Causability Scale Using AI-Based Therapeutic Recommendations for Urological Cancer Patients: A Basis for the Development of a Prospective Comparative Study | Urogenital cancer patients, oncologists | Integration of Large Language Models (LLMs), specifically ChatGPT-4 and Claude 3.5 Sonnet, to generate therapeutic recommendations for urological cancer patients; validation and modification of the System Causability Scale (SCS) to assess the quality of AI recommendations compared to those from multidisciplinary tumor boards (MTBs) | Both the original SCS and its modified version (mSCS) demonstrated strong validity, reliability (Cohen’s K > 0.74), and internal consistency (Cronbach’s Alpha > 0.9), with the mSCS showing superior performance (p < 0.01). Two Delphi processes were used to select the LLMs and set an acceptable non-inferiority margin between AI and MTB recommendations. This work establishes the methodological prerequisites for the upcoming CONCORDIA trial, which will compare blinded mSCS assessments of MTB versus LLM recommendations using 110 urological cancer scenarios. | Ethical: • The study is ethics-approved, but real patient cases were not used due to data privacy regulations, necessitating the creation of realistic simulated cases.  Consent: • Limitations include potential issues when LLM recommendations lack clinical nuance or specificity, raising questions about informed consent for AI-generated treatment advice in complex scenarios.nal SCS and its modified version (mSCS) demonstrated strong validity, reliability (Cohen’s K > 0.74), and internal consistency (Cronbach’s Alpha > 0.9), with the mSCS showing superior performance (p < 0.01). Two Delphi processes were used to select the LLMs and set an acceptable non-inferiority margin between AI and MTB recommendations. This work establishes the methodological prerequisites for the upcoming CONCORDIA trial, which will compare blinded mSCS assessments of MTB versus LLM recommendations using 110 urological cancer scenarios. |
| Curia 2021 | Cervical cancer risk prediction with robust ensemble and explainable black boxes method | Female patients at risk for cervical cancer | Development of a robust ensemble machine learning model for predicting cervical cancer risk. The model integrates advanced ensemble techniques with explainable AI tools (e.g., LIME and Shapley) to provide transparent and interpretable risk predictions. | The proposed model demonstrates promising accuracy and sensitivity in classifying cervical cancer risk. Its explainable outputs are designed to offer clear, understandable recommendations that can be integrated into clinical decision-making, thereby aiding therapeutic planning and patient management. | Ethical: • Need for transparency and interpretability to overcome the “black-box” nature of advanced ML methods.  Legal: • Balancing data privacy and protection of intellectual property with the requirement for openness in clinical decision-making; ensuring that clinicians can be held accountable for AI-assisted decisions. |
| Parikh 2022 | Clinician perspectives on machine learning prognostic algorithms in the routine care of patients with cancer: a qualitative study | 29 oncology clinicians (19 physicians, 10 advanced practice providers) from 6 practice sites (1 tertiary, 5 community) in the USA; 14 had previous exposure to a machine learning prognostic algorithm | Machine learning prognostic algorithms designed to predict mortality risk in cancer, intended to prompt advance care planning and validate clinical intuition | Clinicians recognized the utility of such algorithms for confirming their own prognostic judgments and initiating discussions about patient goals and preferences. - They expressed concerns about the accuracy of algorithm predictions, particularly due to variability in patient responses and potential data quality issues. - There was notable apprehension regarding over-reliance on these tools, which could reduce the human element in care. - Tolerance for false positive versus false negative predictions varied significantly among participants. | Ethical: • Concerns about the ethical implications of disclosing mortality predictions to patients, including potential mental harm and misinterpretation (e.g., the risk that a high mortality prediction might unduly dissuade clinicians from offering beneficial therapies).  Legal: • Uncertainty regarding accountability and liability if algorithm-driven decisions result in adverse outcomes, given that clinical judgment is partly supplanted by algorithm outputs.  Consent: • The need for transparent communication about the use and limitations of prognostic algorithms, ensuring patients are adequately informed about how these tools influence their care and that their consent is obtained under clear, understandable terms. |
| Tan 2021 | Personalised, Rational, Efficacy-Driven Cancer Drug Dosing via an Artificial Intelligence SystEm (PRECISE): A Protocol for the PRECISE CURATE.AI Pilot Clinical Trial | Adults with metastatic solid tumours and elevated baseline tumour marker levels, planned for palliative-intent, capecitabine-based chemotherapy in Singapore | A small-data, personalised dosing platform that uses individual tumour marker levels and administered drug doses to generate efficacy-driven chemotherapy dosing recommendations | The primary outcome is the proportion of participants in whom CURATE.AI can be successfully applied to provide personalised dosing. Secondary outcomes include the timeliness of dose recommendations, adherence to CURATE.AI-recommended doses by both participants and physicians, and the proportion of clinically significant dose changes. The pilot aims to gather key feasibility data to inform a future RCT, potentially improving therapeutic decision-making in precision oncology by optimising drug dosing. | Ethical & Legal: • Patient data will be de-identified via unique patient numbers (UPNs) and stored in a REDCap database on password-protected computers, in compliance with the Personal Data Protection Act 2012. • Only authorised personnel will have access, and data will be maintained for a minimum of 6 years as per ICH GCP guidelines. As a clinical trial, it adheres to ethical committee approval, requiring informed consent from participants and compliance with local regulatory standards. Standard challenges—such as ensuring patient data privacy, maintaining transparency in AI-driven dosing recommendations, and safeguarding patient safety—are implicitly managed within the trial’s ethical framework. Consent: • Written consent will be obtained per SGGCP guidelines and the Declaration of Helsinki. Patients will receive comprehensive study information in an understandable language and are informed of their right to withdraw at any time without penalty. |
| Ng 2023 | Concordance of a decision algorithm and multidisciplinary team meetings for patients with liver cancer—a study protocol for a randomized controlled trial | Patients with hepatobiliary tumors meeting inclusion criteria for tumor board discussion at a single center | ADBoard (Therapeutic Assistance and Decision algorithms for hepatobiliary tumor Boards) aims to improve the MDM process for patients with liver cancer by using different artificial intelligence (AI) methods from the fields of natural language processing (NLP) and machine learning (ML). | The study hypothesizes that ADBoard will yield high concordance with conventional MDM recommendations (target Cohen’s kappa ≥ 0.75), improve the completeness of patient information presented, and enhance the explainability of decision-making protocols (as measured by the System Causability Scale). It is anticipated that ADBoard will streamline the MDM process by reducing administrative workload and allowing clinicians to focus on more complex cases. | Ethical: • Ensuring that automated data extraction accurately captures all relevant patient information without compromising patient safety.  Legal: • Obtaining the proper permissions and network access (e.g., from external partners like DFKI) to access the Health Data Platform in compliance with data protection regulations.  Consent: • Securing informed consent for the use of patient data and maintaining confidentiality when integrating data into the ADBoard system; addressing limitations in capturing subjective or psychosocial factors not documented in the hospital information system. |
| Masiero 2023 | A Machine Learning Model to Predict Patients’ Adherence Behavior and a Decision Support System for Patients With Metastatic Breast Cancer: Protocol for a Randomized Controlled Trial | 100 patients with metastatic breast cancer recruited at the European Institute of Oncology (IEO); 50 patients assigned to the experimental group (exposed to the intervention) and 50 to the control group | A web-based Decision Support System (DSS) named TREAT, featuring four sections (Metastatic Breast Cancer, Adherence to Cancer Therapies, Promoting Adherence, and My Adherence Diary) integrated with a machine learning–based web application. The system predicts risk factors for nonadherence by analyzing physical, psychological, social, and behavioral variables, as well as quality-of-life data collected via standardized self-reports and weekly medication diaries. | Primary Outcome: Evaluate the effectiveness of the DSS and ML application in promoting adherence to oral anticancer treatments. Secondary Outcome: Collect new predictive variables that could refine the ML model for predicting adherence behavior. While recruitment began in May 2023 (expected to conclude December 2023), the anticipated impact is an improvement in medication adherence, which is expected to positively influence clinical outcomes and reduce the economic burden of nonadherence. | Ethical:  • The study is approved by the IEO ethics committee (R1786/22-IEO1907) and adheres to relevant ethical, legal, and data protection regulations.  Legal  • Patient data, including self-reports and medication diaries, are collected via secure, web-based systems with appropriate de-identification measures.ccording to the General Data Protection Regulation (Regulation EU, 2016/679) Consent: • Written informed consent is obtained following ethical guidelines, ensuring patients understand study procedures and their rights, including the right to withdraw without penalty. |
| Tzelves 2022 | Artificial intelligence supporting cancer patients across Europe—The ASCAPE project | Breast and prostate cancer survivors from four European study sites | AI platform integrating patient data (from medical records, questionnaires, apps, wearables) to predict QoL issues and suggest tailored interventions for cancer rehabilitation | Aims to improve QoL by enabling personalized follow-up strategies, enhancing patient engagement, and informing clinician decision-making regarding supportive care | Ethical: The ASCAPE trial ensures ethical integrity by implementing only non-invasive interventions that align with standard clinical practice and are applied through shared decision-making. This guarantees patient autonomy and eliminates the risk of direct physical harm from AI-supported follow-up care. Additionally, the project places a strong emphasis on transparency and respect for participants by clearly informing them about the study's aims and procedures. Participants retain full autonomy, including the right to withdraw consent at any time through an accessible and well-defined process.  Legal: The ASCAPE project is fully aligned with the General Data Protection Regulation (GDPR), particularly Article 5, by designing its data architecture around principles of lawfulness, fairne ss, and transparency. It ensures that data processing remains purpose-specific, minimized, accurate, and securely retained only for as long as necessary. Consent: Participants will receive clear, accessible information about the research goals, data processing, and their rights. They are entitled to withdraw consent at any time through a straightforward withdrawal procedure,  Furthermore, personal data is never transferred outside the EU without appropriate safeguards, and processing is always based on explicit participant consent. This legal framework guarantees participant rights and compliance with both European and national data protection standards. |
| Lococo 2023 | Lung cancer multi-omics digital human avatars for integrating precision medicine into clinical practice: the LANTERN study | 600 lung cancer patients recruited prospectively across five European centers; involvement of multidisciplinary clinical and scientific stakeholders | Digital Human Avatars (DHA): Integration of clinical variables with multi-omics data (genomic, quantitative imaging, etc.) using advanced AI/ML techniques. | Aims to enhance therapeutic decision-making by improving diagnostic accuracy and personalizing treatment pathways. Expected outcomes include reduced overtreatment and toxicity, shortened recovery and hospitalization times, decreased healthcare costs, and overall improved patient quality of life through precise and efficient clinical models. | Ethical: The LANTERN project addresses key ethical issues such as respect for human dignity, fair distribution of research benefits and burdens, and the protection of participants' rights and interests. Special emphasis is placed on ethical oversight throughout the project lifecycle, particularly concerning the responsible development and use of AI systems in accordance with the Declaration of Helsinki and GDPR. Legal: A comprehensive Data Management Plan (DMP) is implemented to comply with GDPR and adhere to FAIR principles. Challenges include ensuring data privacy and security, obtaining informed consent for multi-source data, managing open data access with strict monitoring, and meeting legal requirements for data governance and regulatory compliance. Consent: All participants in the LANTERN project will be thoroughly informed about the nature, aims, and procedures of the study. Consent will be obtained through a clear, comprehensive process involving a signed informed consent form, ensuring participants understand their rights and the use of their data. |
| Aghamaliyev 2024 | ChatGPT's Gastrointestinal Tumor Board Tango: A limping dance partner? | 115 cases of gastrointestinal malignancies presented at MTBs | ChatGPT 3.5 was used to generate treatment recommendations based on patient data provided by a senior resident; these recommendations were compared with those from MTBs. | ChatGPT provided case-specific treatment recommendations in 81% of cases, with overall treatment strategy concordance at 83% for those cases. However, exact treatment plan concordance was 65%, with lower precision observed for chemotherapy regimens and follow-up protocols. | Ethical: Ethical concerns include the risk of imprecise recommendations impacting patient safety; challenges in replicating exact treatment plans (especially chemotherapy and follow-up) raise issues related to clinical liability and the appropriateness of using ChatGPT directly for clinical decision-making in MTBs. |
| Janbain 2024 | A Machine Learning Approach for Predicting Biochemical Outcome After PSMA-PET-Guided Salvage Radiotherapy in Recurrent Prostate Cancer After Radical Prostatectomy: Retrospective Study | 1029 prostate cancer patients undergoing salvage radiotherapy following PSMA-PET-based assessment for PSA persistence or recurrence (data collected from 13 medical facilities across 5 countries: Germany, Cyprus, Australia, Italy, and Switzerland; median age ~70 years) | Developed a Random Survival Forest (RSF) model to predict freedom from biochemical failure after salvage radiotherapy; utilized k-fold cross-validation for hyperparameter tuning and statistical performance evaluation (Harrell C-index, Brier score); compared RSF with a Cox model and an existing nomogram. | The RSF model demonstrated robust predictive performance (Harrell C-index range: 0.54–0.91) across training, testing, and external validation datasets, outperforming a previously published nomogram; its predictive accuracy may assist clinicians in tailoring treatment decisions for recurrent prostate cancer. | Ethical: The study complied with ethical standards at all participating centers, with formal approval obtained from each institutional ethics board. The retrospective design of the study minimized patient risk, and ethical integrity was maintained by following guidelines for the secondary use of clinical data. Legal: Data handling respected privacy regulations, with deidentification ensuring compliance with data protection norms. Consent: Due to the study’s retrospective nature and existing ethical approval, informed consent was waived. However, original informed consent obtained during primary data collection explicitly allowed for secondary data use, aligning with ethical and legal expectations for consent in observational research. |
| Lazris 2024 | AI-Generated Content in Cancer Symptom Management: A Comparative Analysis Between ChatGPT and NCCN | Cancer patients seeking supportive care for cancer-related symptoms (as represented by NCCN guidelines) | ChatGPT-3.5 was used to generate treatment recommendations for nine cancer-related symptoms by entering queries (e.g., "How can I reduce my cancer-related [symptom]?"). These recommendations were compared with those provided by the National Comprehensive Cancer Network (NCCN) for supportive and palliative care. | Mean percent agreement between ChatGPT and NCCN recommendations was 37.3% (range 16.7%-81.8%). - NCCN provided more specific medication recommendations, whereas ChatGPT occasionally suggested medications not mentioned in NCCN (notably in constipation and diarrhea sections). - Significant differences were observed in word count and readability (Flesch-Kincaid Grade Level), with ChatGPT responses being more concise and having a lower reading level. | Ethical: • Discrepancies between AI-generated content and evidence-based guidelines may mislead patients and affect care quality. Legal: • Potential liability issues if patients act on inaccurate or non-evidence-based recommendations provided by AI. Consent: • Patients may be unaware of the limitations and discrepancies in AI-generated advice versus professional guidelines, raising concerns over informed decision-making. |
| Shimada 2023 | Novel Method for Predicting Nonvisible Symptoms Using Machine Learning in Cancer Palliative Care | 213 end-of-life cancer patients receiving palliative care | Machine learning using decision tree analysis to predict nonvisible symptoms based on patient background data and reported visible symptoms | The model predicted nonvisible symptoms—including pain, dyspnea, fatigue, drowsiness, anxiety, delirium, inadequate informed consent, and spiritual issues—with accuracy ranging from 55.5% to 88.0%, sensitivity from 3.3% to 84.9%, and specificity from 24.1% to 96.7%. This approach has the potential to enhance symptom assessment and management in palliative care by approximating the evaluation capabilities of healthcare professionals. | Ethical: The inclusion of "inadequate informed consent" as a predicted symptom underscores challenges in patient communication and decision-making at end-of-life.  Legal: Although not explicitly discussed, the reliance on AI for symptom assessment in vulnerable populations raises questions regarding accountability and clinical liability.  Consent: Ensuring that AI predictions are interpreted correctly is critical, as misinterpretation might exacerbate issues related to informed consent and patient autonomy in sensitive care settings. |
| Stalp 2024 | Quality of ChatGPT-Generated Therapy Recommendations for Breast Cancer Treatment in Gynecology | 30 breast cancer cases | ChatGPT (version unspecified, likely 3.5) used to generate therapy recommendations for breast cancer treatment; standardized patient details and treatment options were provided as input; outputs were evaluated by oncologists using a structured questionnaire | Overall, ChatGPT’s recommendations were rated as sufficient with minor limitations. - Best performance was observed in the HER2 treatment category with high accuracy. - Primary (non-complicated) cases received more accurate recommendations, especially regarding chemotherapy. - Challenges were noted in complex cases and postoperative scenarios, particularly in providing proper chronological treatment sequences and precise recommendations. | Ethical: • Need to address ethical intricacies in relying on AI-generated treatment advice for patient care. • Balancing AI recommendations with expert clinical insights to ensure patient safety. Legal: • Although not explicitly discussed, safe implementation is implied to be crucial to avoid potential medico-legal liability. Consent: • Ensuring that patients are informed about the use of AI in treatment planning is critical, even if not directly addressed; refining input data and addressing limitations are necessary steps for responsible integration. |
| Hesjedal 2024 | Valuing good health care: How medical doctors, scientists and patients relate ethical challenges with artificial intelligence decision-making support tools in prostate cancer diagnostics to good health care | Three stakeholder groups: • Scientists developing AI decision-support tools for interpreting prostate cancer MRI scans • Medical doctors (MDs) working in prostate cancer care • Prostate cancer patients | AI decision-making support tools in prostate cancer diagnostics (with a focus on how these tools are perceived and evaluated in terms of contributing to "good health care") | • Scientists: Emphasize the need for robust, representative data and rigorous testing/validation of AI models to ensure accuracy and accountability. • MDs: Express optimism about AI’s future potential to enhance diagnostic accuracy while underscoring that AI should support rather than replace human expertise, especially in complex cases. • Patients: Place high trust in MDs and are cautious about AI, relying on professionals to ensure that AI tools are thoroughly tested. | Ethical: • Accountability: Good health care requires that AI-driven decisions be made by identifiable humans who can be held responsible. • Human qualities (ethics/morals): AI lacks inherent ethical attributes, making the transfer of ethical standards from developers to AI critical.  Legal: • Ambiguity in liability: Unclear legal frameworks regarding who is responsible for AI-based decisions, with concerns about potential medico-legal consequences if AI recommendations lead to errors. |

**Supplementary Table S3.** Main findings of included studies
